# Supplementary material for: The effects of a second pregnancy on women’s brain structure and function
Source: Nat Commun. 2026 Feb 19;17:1495. doi: 10.1038/s41467-026-69370-8 (PMC12920768; doi:10.1038/s41467-026-69370-8)
Supplement: Supplementary file 1 — Supplementary Information [file 41467_2026_69370_MOESM1_ESM.pdf]

The effects of a second pregnancy on women's brain structure and function

Supplementary Information

Supplementary Table 1: Cohen's D effect sizes for vertex-wise analyses between groups.

Contrast	Cohen's D
PRG2 - CTR	0.97 – 1.67 (mean: 1.14)
PRG1 - CTR	0.83 – 1.61 (mean: 1.05)
PRG1 – PRG2	0.50 – 0.97 (mean: 0.61)

Note. Range of Cohen's D effect sizes for the three different contrasts in our vertex-wise analyses. PRG2: multiparous women (n = 30) ; PRG1: primiparous women (n = 40); CTR: nulliparous control women (n = 40).

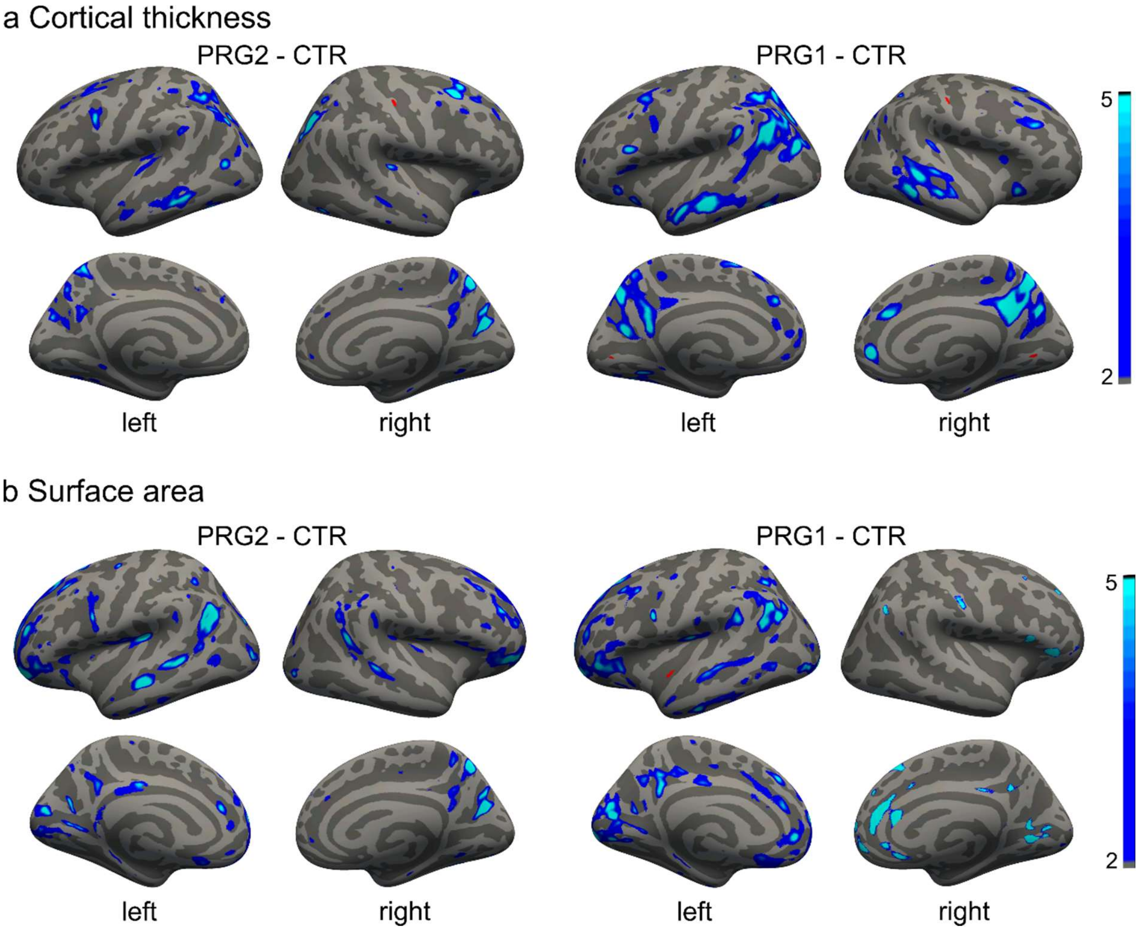

Supplementary Fig. 1: Longitudinal vertex-wise analyses of cortical thickness and surface area changes across pregnancy. Vertex-wise analyses results showing brain areas that decrease in cortical thickness (a) or surface area (b) across pregnancy in women pregnant of their second child (PRG2; left; n=30) and women pregnant of their first child (PRG1; right; n=40) compared to control women (CTR; n=40).

Values represent -log(FDR corrected p-value), with 2 being p = 0.01. Source data are provided as a Source Data file.

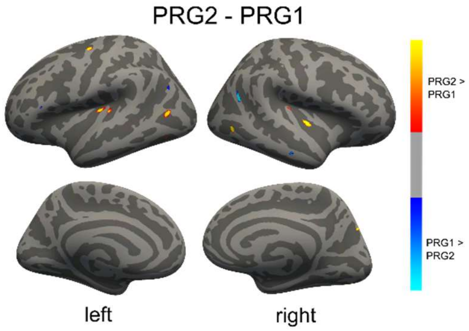

Supplementary Fig. 2: Vertex-wise analyses comparing brain changes across a first and second pregnancy. Vertex-wise analyses results showing brain areas that decrease in volume across pregnancy in women pregnant of their second child (PRG2; n=30) compared to women pregnant of their first child (PRG1; n=40). Coloured vertices are significant vertices after 1000 permutations, using p < 0.01 at a vertex-level, corrected for measuring across the left and right hemisphere. Source data are provided as a Source Data file.

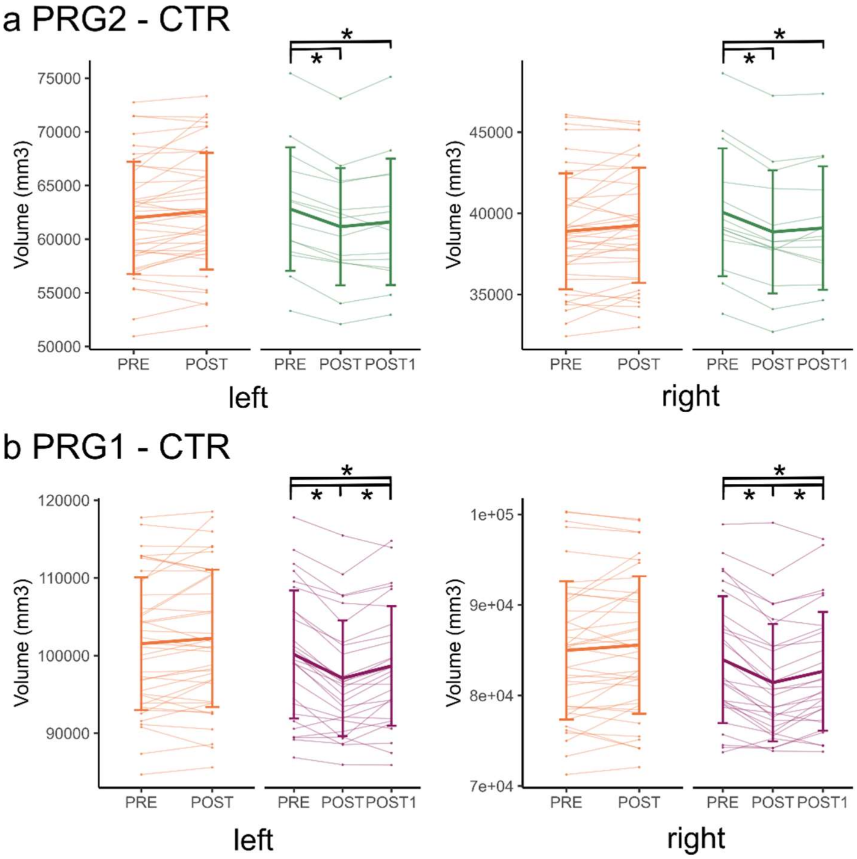

Supplementary Fig. 3: Brain volumetric decreases remain into the late postpartum period in multiparous and primiparous women. Regions-of-interest were the FDR-corrected significant vertices from the vertex-wise analyses comparing PRE and POST from PRG2 and CTR (a) and PRG1 and CTR (b). As

a result the ROIs of which we extracted the volumes were different for the PRG2-CTR comparison and PRG1-CTR comparison. Data of control women are presented in orange, second-time mothers in green and first-time mothers in purple. Extracted total volumes of these significant vertices are shown in mm^3 for the three timepoints: before pregnancy (PRE), in the early postpartum period (POST) and the late postpartum period (POST1). Since the late postpartum session could only be performed in a subset of women (PRG2: n=14; PRG1: n=28), these plots only show complete datasets of multiparous and primiparous women, next to all control women (CTRL; n=40). * $p < 0.001$ for paired t-tests within groups. Source data are provided as a Source Data file.

Supplementary Table 2: Extracted brain volumes at different time points for multiparous and primiparous women in complete datasets

Multiparous (PRG2)

Volume (mm^3)			Paired t-tests / Wilcoxon signed rank tests		
	Left	Right		Left	Right
PRE	62806 ± 5754	40068 ± 3941	PRE-POST	$t(13) = 9.62, p = 2.81e^{-07}$	$t(13) = 9.87, p = 2.10e^{-07}$
POST	61162 ± 5462	38858 ± 3795	PRE-POST1	$t(13) = 5.62, p = 8.08e^{-05}$	$t(13) = 6.28, p = 2.83e^{-05}$
POST1	61614 ± 5891	39099 ± 3804	POST-POST1	$t(13) = -1.81, p = 0.09$	$t(13) = -1.48, p = 0.16$

Primiparous (PRG1)

Volume (mm^3)			Paired t-tests / Wilcoxon signed rank tests		
	Left	Right		Left	Right
PRE	100154 ± 8241	83957 ± 7007	PRE-POST	$t(27) = 7.70, p = 2.80e^{-08}$	$t(27) = 7.33, p = 6.99e^{-08}$
POST	97088 ± 7450	81425 ± 6482	PRE-POST1	$t(27) = 4.25, p = 0.0002$	$t(27) = 4.09, p = 0.0003$
POST1	98677 ± 7707	82670 ± 6558	POST-POST1	$t(27) = -4.69, p = 6.86^{e-05}$	$t(27) = -4.41, p = 0.0001$

Note. Extracted brain volumes in mm^3 (mean ± standard deviation) from the vertex-wise analyses in the left and right hemisphere in multiparous (PRG2; n=14) and primiparous (PRG1; n=28) women at three time points: before pregnancy (PRE), in the early postpartum period (POST) and late postpartum period (POST1). The vertex-wise comparisons were between PRG2 and control (CTR; n=40) and between PRG1 and CTR, and as a result the ROIs of which we extracted the volumes were different for the PRG2 and PRG1 group. Two-sided paired t-tests are performed between timepoints in each group. Only complete datasets are considered, resulting in 14 multiparous and 28 primiparous women.

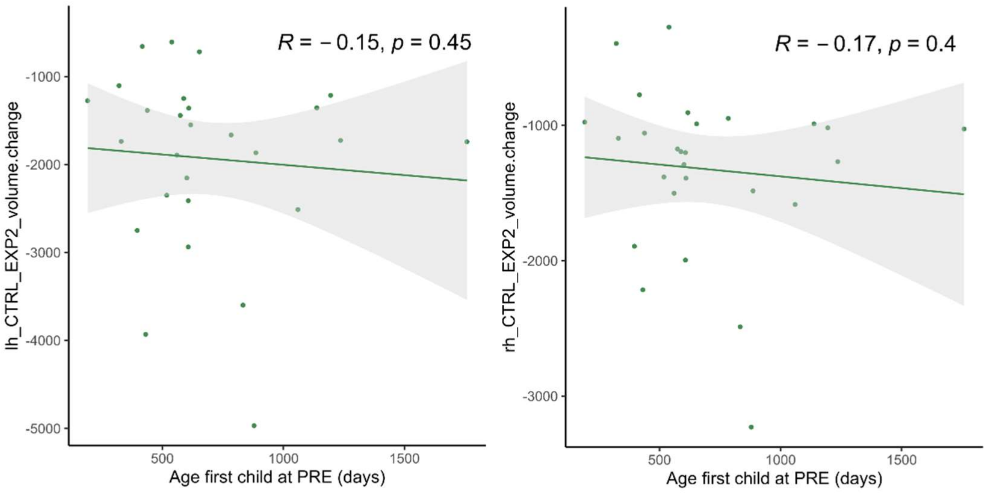

Supplementary Fig. 4: Correlation between age of the first child at PRE session and volumetric brain changes across a second pregnancy in the left and right hemisphere. Age of first child at the PRE session is given in days, and extracted brain volume changes in mm^3 inside the area of change across a second pregnancy. Individual data points are shown, and the corresponding Spearman correlation value (R) and p-value are plotted. Source data are provided as a Source Data file.

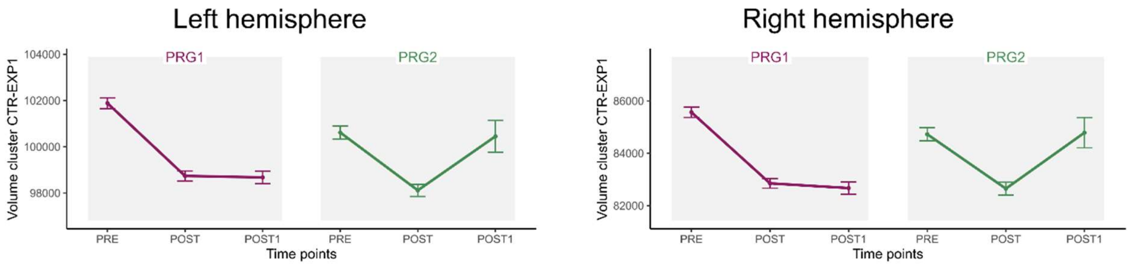

Supplementary Fig. 5: Grey matter volumes across a first and second pregnancy. Extracted brain volumes in mm^3 inside the area of change across a first pregnancy, also shown across a second pregnancy in the left and right hemisphere on three time points: before pregnancy (PRE), in the early postpartum period (POST) and the late postpartum period (POST1). The POST1 measurement is performed in a subset of women (PRG1: n=28; PRG2: n=14). The error bars represent the standard error of the mean. Source data are provided as a Source Data file.

Supplementary Table 3. Measures of maternal behaviour and mental health status across a first and second pregnancy.

Time	Questionnaire	PRG2	PRG1	Group difference
During pregnancy	MAAS	76.79 ± 5.69	79.60 ± 6.89	F(1,61) = 3.02, p = 0.09
	PAI	59.25 ± 10.10	62.11 ± 7.91	F(1,61) = 1.59, p = 0.21
	Nesting Behaviour	10.11 ± 3.28	10.63 ± 2.81	F(1,61) = 0.46, p = 0.50
	K10	9.07 ± 6.89	7.63 ± 4.47	F(1,61) = 1.01, p = 0.32
	EPDS	12.29 ± 2.48	11.17 ± 2.05	F(1,61) = 3.82, p = 0.06
Early postpartum	MPAS	80.27 ± 7.73	81.28 ± 6.52	F(1,65) = 0.34, p = 0.57
	PBQ	11.36 ± 7.71	11.23 ± 7.58	F(1,65) = 0.004, p = 0.95
	K10	6.68 ± 6.12	7.21 ± 6.14	F(1,65) = 0.12, p = 0.73
	EPDS	11.71 ± 2.07	11.21 ± 2.25	F(1,65) = 0.89, p = 0.35

Note. Questionnaire results (mean ± standard deviation) in multiparous (PRG2) and primiparous (PRG1) women, acquired during pregnancy (in the third trimester; PRG2: n=28, PRG1: n=36) and the early postpartum period (PRG2: n=28, PRG1: n=39). Results of group comparisons using a conventional right-tailed ANOVA are shown. MAAS: Maternal Antenatal Attachment Scale; PAI: Prenatal Attachment Inventory; Nesting behaviour: Nesting Behaviour Questionnaire; EPDS: Edinburgh Postnatal Depression Score; MPAS: Maternal Postnatal Attachment Scale; PBQ: Postpartum Bonding Questionnaire.

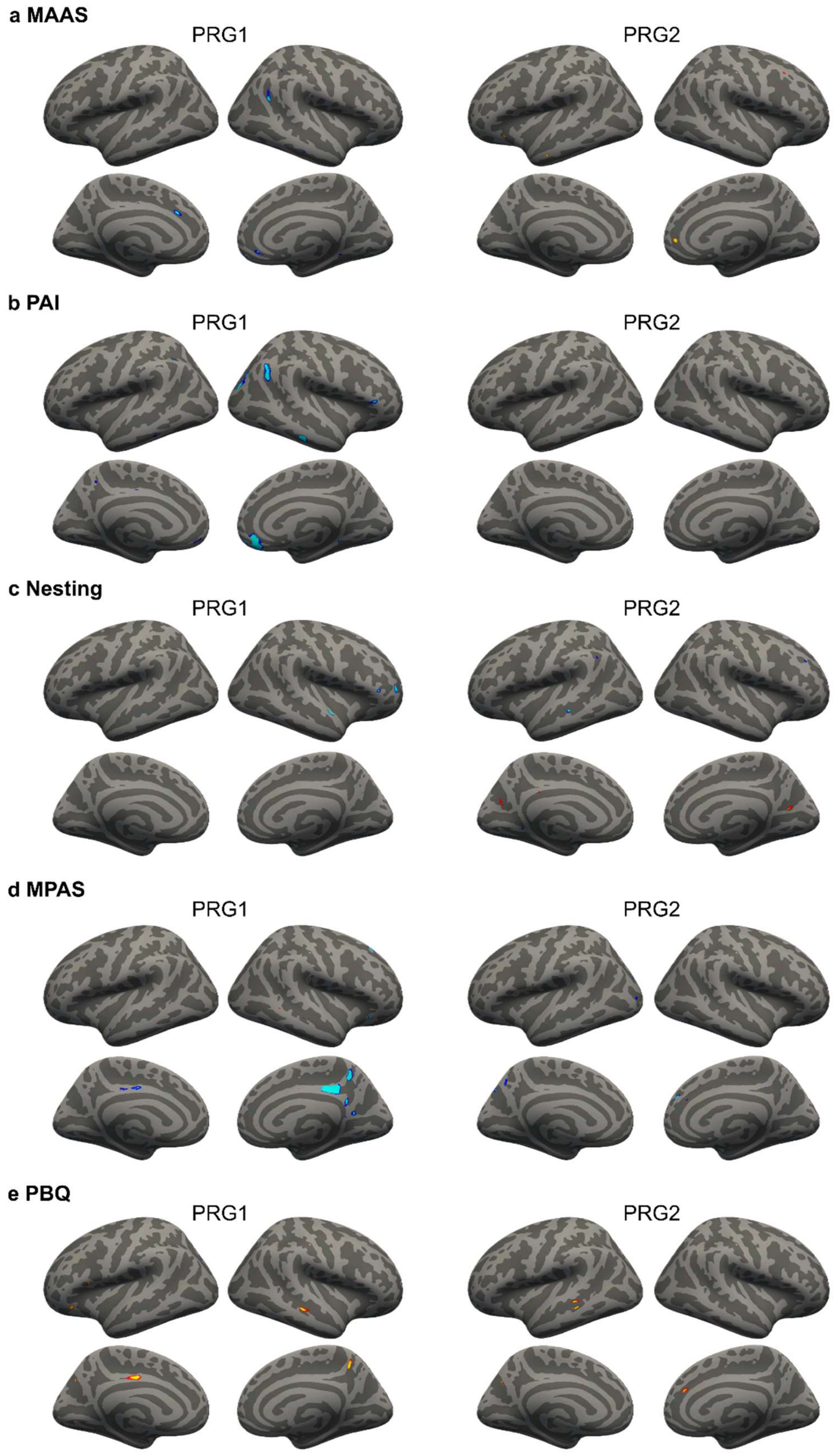

Supplementary Fig. 6: Vertex-wise analyses correlating volumetric brain changes with maternal behaviour during pregnancy and in the postpartum period. Vertex-wise analyses results showing brain areas with a significant correlation between the volumetric brain changes across a first (PRG1) or second (PRG2) pregnancy and measures of maternal behaviour during pregnancy (a, b, c) and in the early

postpartum period (d, e). MAAS: Maternal Antenatal Attachment Scale; PAI: Prenatal Attachment Inventory; Nesting behaviour: Nesting Behaviour Questionnaire; MPAS: Maternal Postnatal Attachment Scale; PBQ: Postpartum Bonding Questionnaire. Red colours represent positive correlations, whereas blue colours represent negative correlations between volumetric brain changes and maternal behaviour. Source data are provided as a Source Data file.

Supplementary Table 4: Number of significant vertices and mean correlation between volumetric change and maternal behaviour across a first and second pregnancy.

Questionnaire	Group	Significant vertices	Correlation
MAAS	PRG1	907	-0.50 ± 0.03
	PRG2	125	0.53 ± 0.03
PAI	PRG1	2005	-0.48 ± 0.03
	PRG2	0	
Nesting	PRG1	337	-0.50 ± 0.04
	PRG2	178	- 0.51 ± 0.03
		160	0.51 ± 0.01
MPAS	PRG1	1506	-0.48 ± 0.04
	PRG2	283	-0.51 ± 0.03
PBQ	PRG1	723	0.47 ± 0.02
	PRG2	202	0.51 ± 0.02

Note. Extracted number of vertices (sum of left and right hemisphere) that showed a significant correlation between volumetric brain changes and maternal behavioural scales across a first (PRG1, n = 40) or a second (PRG2 n = 30) pregnancy, and the mean ± standard deviation of these correlations. MAAS: Maternal Antenatal Attachment Scale; PAI: Prenatal Attachment Inventory; Nesting behaviour: Nesting Behaviour Questionnaire; MPAS: Maternal Postnatal Attachment Scale; PBQ: Postpartum Bonding Questionnaire. We excluded correlations that consisted of less than 50 significant vertices.

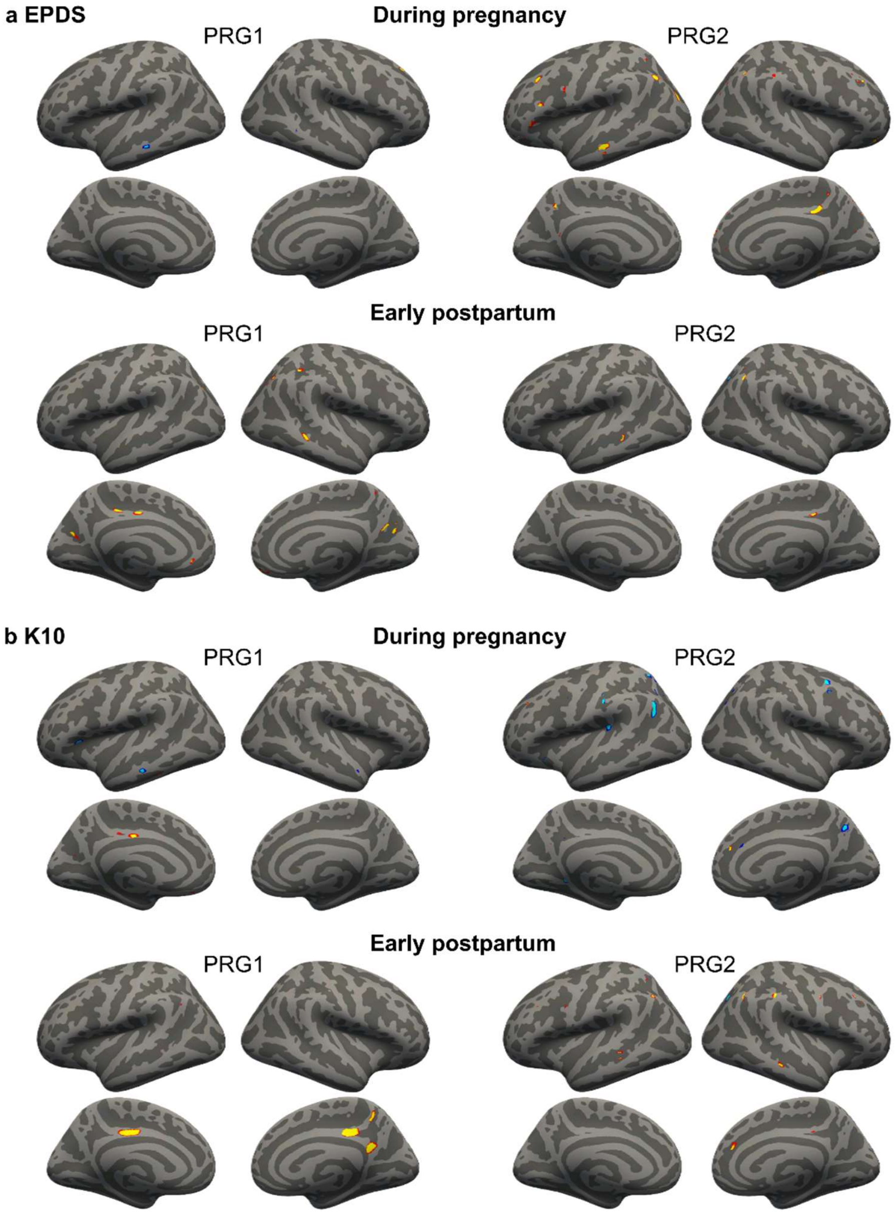

Supplementary Fig. 7: Vertex-wise analyses correlating volumetric brain changes across pregnancy with maternal mental health during pregnancy and in the early postpartum period. Vertex-wise analyses results showing brain areas with a significant correlation between the volumetric brain changes across a first (PRG1, n = 40) or a second (PRG2, n = 30) pregnancy and measures of maternal mental health, measured during pregnancy (in the third trimester) or in the early postpartum period. We measured levels of depression with the Edinburgh Postnatal Depression Score (EPDS)(a) and levels of psychological distress with the K10 questionnaire (b). Red colours represent positive correlations, whereas blue colours represent negative correlations between volumetric brain changes and maternal mental health. Source data are provided as a Source Data file.

Supplementary Table 5: Number of significant vertices and mean correlation between volumetric change and maternal mental health across a first and second pregnancy.

Questionnaire	Group	Significant vertices	Correlation
EPDS during pregnancy	PRG1	79	-0.47 ± 0.01
	PRG2	2051	0.55 ± 0.05
EPDS early postpartum	PRG1	1200	0.47 ± 0.03
	PRG2	78	-0.52 ± 0.03
		375	0.55 ± 0.04
K10 during pregnancy	PRG1	159	- 0.47 ± 0.02
		203	0.48 ± 0.02
	PRG2	1365	-0.53 ± 0.04
		160	0.51 ± 0.03
K10 early postpartum	PRG1	1537	0.49 ± 0.04
	PRG2	123	-0.55 ± 0.04
		592	0.52 ± 0.03

Note. Extracted number of vertices (sum of left and right hemisphere) that showed a significant correlation between volumetric brain changes across a first (PRG1, n = 40) and second (PRG2, n = 30) pregnancy and maternal mental health scales measured in the third trimester of pregnancy and the early postpartum period, and the mean ± standard deviation of these correlations. EPDS: Edinburgh Postnatal Depression Score; K10: measure of psychological distress. We excluded correlations that consisted of less than 50 significant vertices.

Supplementary Table 6: Cluster-wise correction of vertex-wise analyses comparing brain volume between multiparous and control women in left hemisphere.

Left	Max	Size (mm^2)	MNI X	MNI Y	MNI Z	CWP	Annotation
1	8.65	14774.22	-6.2	-70.4	36.4	0.0002	Precuneus
2	7.76	6152.22	-8.0	59.5	23.4	0.0002	Superior frontal
3	7.43	2735.05	-43.6	29.1	-14.2	0.0002	Pars orbitalis
4	7.85	1049.74	-53.0	-44.2	30.6	0.0002	Supramarginal
5	6.57	834.68	-6.0	28.4	-18.7	0.0002	Medial Orbitofrontal
6	7.10	762.45	-32.6	-32.0	18.3	0.0002	Insula
7	6.73	662.99	-44.2	1.8	27.4	0.0002	Precentral
8	5.81	388.09	-16.9	-37.4	-11.2	0.0002	Parahippocampal
9	4.67	256.28	-30.6	-13.1	58.3	0.0008	Precentral
10	4.08	200.24	-34.0	47.9	8.5	0.0026	Rostral middle frontal
11	4.41	196.84	-30.2	-82.6	4.1	0.0028	Lateral Occipital
12	5.83	192.84	-9.9	33.2	18.1	0.0032	Rostral anterior cingulate
13	4.44	166.77	-52.3	-22.0	38.6	0.0076	Postcentral
14	4.19	135.67	-8.9	0.9	54.8	0.025	Superior frontal

Note. Results from cluster-wise correction of vertex-analyses between multiparous (n=30) and control women (n=40) in the left hemisphere. Max = maximum -log10(p value) in the cluster; CWP = cluster wise p-value.

Supplementary Table 7: Cluster-wise correction of vertex-wise analyses comparing brain volume between multiparous and control women in right hemisphere.

Right	Max	Size (mm^2)	MNI X	MNI Y	MNI Z	CWP	Annotation
1	7.84	10404.83	31.6	17.3	46.3	0.0002	Caudal middle frontal
2	8.74	4141.46	21.1	-71.0	19.7	0.0002	Precuneus
3	6.72	3358.97	34.8	-76.6	30.7	0.0002	Inferior parietal
4	5.81	629.63	61.2	-26.4	-12.4	0.0002	Middle temporal
5	6.35	625.07	33.0	-57.6	-15.0	0.0002	Fusiform
6	6.62	534.61	21.1	-59.3	-7.1	0.0002	Lingual
7	4.42	497.85	16.3	-89.3	18.5	0.0002	Lateral occipital
8	5.13	486.55	45.3	-22.5	8.1	0.0002	Transverse temporal
9	4.87	425.83	8.0	59.1	-7.9	0.0002	Medial orbitofrontal
10	6.38	284.01	20.9	-83.5	-10.6	0.0002	Lateral occipital
11	6.16	199.87	49.0	-60.6	-3.9	0.0020	Inferior temporal
12	6.12	198.00	22.7	-19.8	-24.7	0.0022	Parahippocampal
13	4.70	133.48	49.4	4.4	-23.7	0.025	Superior temporal
14	4.38	117.44	40.3	-33.7	60.3	0.042	Postcentral

Note. Results from cluster-wise correction of vertex-analyses between multiparous (n=30) and control women (n=40) in the right hemisphere. Max = maximum -log10(p value) in the cluster; CWP = cluster wise p-value.

Supplementary Table 8: Cluster-wise correction of vertex-wise analyses comparing brain volume between primiparous and control women in left hemisphere.

Left	Max	Size (mm^2)	MNI X	MNI Y	MNI Z	CWP	Annotation
1	9.92	18703.30	-5.4	-70.8	38.2	0.0002	Precuneus
2	10.00	15159.38	-48.9	19.7	18.8	0.0002	Pars Opercularis
3	5.05	130.51	-31.1	19.7	0.6	0.032	Insula
4	4.10	127.86	-31.2	-48.9	61.8	0.035	Superior parietal

Note. Results from cluster-wise correction of vertex-analyses between primiparous (n=40) and control women (n=40) in the left hemisphere. Max = maximum -log10(p value) in the cluster; CWP = cluster wise p-value.

Supplementary Table 9: Cluster-wise correction of vertex-wise analyses comparing brain volume between primiparous and control women in right hemisphere

Left	Max	Size (mm^2)	MNI X	MNI Y	MNI Z	CWP	Annotation
1	9.91	14089.32	30.3	23.0	-15.7	0.0002	Lateral orbitofrontal
2	9.78	10032.68	10.2	-55.2	31.3	0.0002	Precuneus
3	9.17	3696.55	57.0	-28.1	-16.9	0.0002	Middle temporal
4	6.75	2672.89	34.6	-56.4	-15.3	0.0002	Fusiform
5	5.77	161.43	35.3	-88.1	-1.6	0.008	Lateral occipital
6	4.42	125.90	25.3	-18.3	-29.2	0.033	Parahippocampal
7	4.53	116.65	40.3	-14.5	21.9	0.040	Postcentral

Note. Results from cluster-wise correction of vertex-analyses between primiparous (n=40) and control women (n=40) in the right hemisphere. Max = maximum -log10(p value) in the cluster; CWP = cluster wise p-value.

Supplementary Table 10: Quantification of spatial similarity between grey matter volumetric areas affected in both first and second pregnancies and functional brain networks

Network	Volume network (mm³)	Observed similarity (mm³)	Observed similarity (% network)	Observed similarity (%overlap area)	Expected similarity (mm³)	Observed/ Expected similarity
Visual	73167	6017	8.22	6.50	6899	0.88
Somato-sensory	73452	1116	1.52	1.21	5163	0.16
Dorsal attention	54968	4456	8.11	4.82	5377	0.86
Ventral Attention	57247	5929	10.36	6.41	6066	1.10
Limbic	64580	6177	9.56	6.68	7390	1.02
Fronto-parietal	78676	8454	10.75	9.14	12090	1.14
Default mode	128726	22743	17.67	24.58	6899	1.88

Note. The intersection of the overlap ROI (clusters changing across both a first and second pregnancy) were quantified with the seven resting-state networks of Yeo[1] (Column 'Observed similarity'). The observed similarity was subsequently reported as percentage of the size of the network (column 'Observed similarity (%network)) and as percentage of the size of the overlap area (column 'Observed similarity (% overlap area)). We calculated the expected similarity based on a random distribution across grey matter voxels in the brain (column 'Expected similarity') and determined the ratio of observed/expected similarity (column 'Observed/Expected similarity').

Supplementary Table 11: Quantification of spatial similarity between grey matter volumetric areas only affected in a first pregnancy and functional brain networks

Network	Volume network (mm³)	Observed similarity (mm³)	Observed similarity (% network)	Observed similarity (%CTR-PRG1 area)	Expected similarity (mm³)	Observed/ Expected similarity
Visual	73167	1728	2.36	3.32	3865	0.45
Somato-sensory	73452	2025	2.76	3.89	3880	0.52
Dorsal attention	54968	2183	3.97	4.20	2903	0.75
Ventral Attention	57247	4011	7.01	7.71	3024	1.33
Limbic	64580	3864	5.98	7.43	3411	1.13
Fronto-parietal	78676	6019	7.65	11.57	4156	1.45
Default mode	128726	12116	9.41	23.28	6799	1.78

Note. The intersection of the only CTR-PRG1 ROI (clusters changing specifically in a first pregnancy) were quantified with the seven cognitive networks of Yeo[1] (Column 'Observed similarity'). The observed similarity was subsequently reported as percentage of the size of the network (column 'Observed similarity (%network)) and as percentage of the size of the only CTR-PRG1 area (column 'Observed similarity (% CTR-PRG1 area)). We calculated the expected similarity based on a random distribution across grey matter voxels in the brain (column 'Expected similarity') and determined the ratio of observed/expected similarity (column 'Observed/Expected similarity').

Supplementary Table 12: Quantification of spatial similarity between grey matter volumetric areas only affected in a second pregnancy and functional brain networks

Network	Volume network (mm^3)	Observed similarity (mm^3)	Observed similarity (% network)	Observed similarity (%CTR-PRG2 area)	Expected similarity (mm^3)	Observed/ Expected similarity
Visual	73167	1634	2.23	6.57	1846	0.89
Somato-sensory	73452	2191	2.98	8.82	1853	1.18
Dorsal attention	54968	2104	3.83	8.47	1387	1.52
Ventral Attention	57247	1699	2.97	6.84	1444	1.18
Limbic	64580	880	1.36	3.54	1629	0.54
Fronto-parietal	78676	2073	2.63	8.34	1985	1.04
Default mode	128726	2282	1.77	9.18	3248	0.70

Note. The intersection of the only CTR-PRG2 ROI (clusters changing specifically in a second pregnancy) were quantified with the seven cognitive networks of Yeo[1] (Column 'Observed similarity'). The observed similarity was subsequently reported as percentage of the size of the network (column 'Observed similarity (%network)) and as percentage of the size of the only CTR-PRG2 area (column 'Observed similarity (% CTR-PRG2 area)). We calculated the expected similarity based on a random distribution across grey matter voxels in the brain (column 'Expected similarity') and determined the ratio of observed/expected similarity (column 'Observed/Expected similarity').

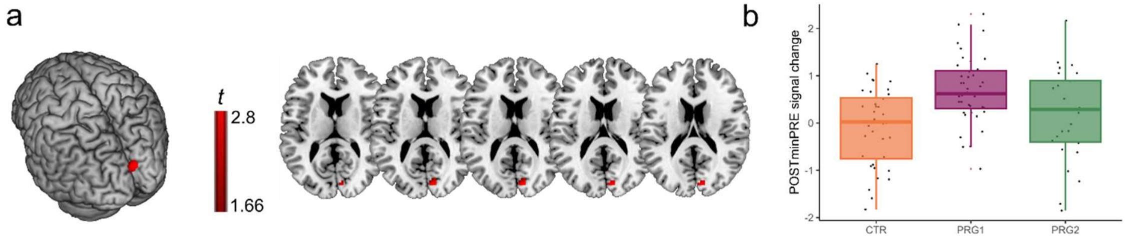

Supplementary Fig. 8: Default Mode Network coherence change from pre to post pregnancy. Significant differences in within-network coherence changes across a first and second pregnancy using the default mode network ROI mask (based on our previous results[2]) (a). Results are shown with p<0.05 after FWE-correction, as surface map (left) and on axial slices (right). The results are characterized by an increase in DMN coherence present across a first pregnancy (MNI coordinates (x y z) = 6 -84 18, T=5.55, p = 0.001 FWE-corrected), but not across a second pregnancy (MNI coordinates (x y z) = 9 -78 15, T=1.68, p = 0.080 FWE-corrected). (b) Extracted signal changes from the cluster shown in a, in the control group (CTR; n=36), primiparous group (PRG1; n=40) and multiparous group (PRG2; n=27), calculated by subtracting the PRE pregnancy signal from the POST pregnancy signal (POSTminPRE)(b). Boxplots show the median, the first (bottom) and third (top) quartile and 1.5 * interquartile range. Underneath, individual data points are shown. Source data are provided as a Source Data file.

Supplementary Table 13: Differences in between network connectivity changes across a second pregnancy compared to nulliparous control women.

	Visual 2	Visual 3	Sensori motor	Visual 1	DMN	Cogn Lang	Audi-tory	Perc Pain	Cere-bellum	Exe-cutive
Visual 2		F=0.20 p=0.87	F=5.91 p=0.10	F=2.78 p=0.33	F=0.20 p=0.87	F=0.57 p=0.74	F=5.03 p=0.14	F=0.71 p=0.73	F=0.14 p=0.90	F=3.28 p=0.28
Visual 3	F=0.20 p=0.87		F=1.29 p=0.56	F=0.66 p=0.74	F=1.15 p=0.59	F=0.15 p=0.89	F=0.87 p=0.66	F=0.30 p=0.81	F=0.17 p=0.89	F=0.10 p=0.91
Sensori motor	F=5.91 p=0.10	F=1.29 p=0.56		F=0.98 P=0.64	F=0.10 p=0.91	F=0.49 p=0.74	F=1.95 p=0.42	F=0.45 p=0.74	F=0.21 p=0.87	F=0.08 p=0.93
Visual 1	F=2.78 p=0.33	F=0.66 p=0.74	F=0.98 P=0.64		F=0.12 p=0.90	F=1.18 p=0.59	F=3.81 p=0.23	F=0.44 p=0.74	F=0.01 p=0.96	F=6.64 p=0.07
DMN	F=0.20 p=0.87	F=1.15 p=0.59	F=0.10 p=0.91	F=0.12 p=0.90		F=3.43 p=0.27	F=0.06 p=0.93	F=2.43 p=0.35	F=5.54 p=0.12	F=1.67 p=0.46
Cogn-Lang	F=0.57 p=0.74	F=0.15 p=0.89	F=0.49 p=0.74	F=1.18 p=0.59	F=3.43 p=0.27		F=0.34 p=0.80	F<0.01 p=0.96	F=2.37 p=0.35	F=1.11 p=0.59
Audi-tory	F=5.03 p=0.14	F=0.87 p=0.66	F=1.95 p=0.42	F=3.81 p=0.23	F=0.06 p=0.93	F=0.34 p=0.80		F=0.53 p=0.74	F=0.31 p=0.81	F=5.30 p=0.12
Perc-Pain	F=0.71 p=0.73	F=0.30 p=0.81	F=0.45 p=0.74	F=0.44 p=0.74	F=2.43 p=0.35	F<0.01 p=0.96	F=0.53 p=0.74		F=0.54 p=0.74	F=0.04 p=0.94
Cere-bellum	F=0.14 p=0.90	F=0.17 p=0.89	F=0.21 p=0.87	F=0.01 p=0.96	F=5.54 p=0.12	F=2.37 p=0.35	F=0.31 p=0.81	F=0.54 p=0.74		F=0.95 p=0.64
Exe-cutive	F=3.28 p=0.28	F=0.10 p=0.91	F=0.08 p=0.93	F=6.64 p=0.07	F=1.67 p=0.46	F=1.11 p=0.59	F=5.30 p=0.12	F=0.04 p=0.94	F=0.95 p=0.64	

Note. We compared between-network connectivity changes (from PRE to POST) between all identified 10 functional networks in multiparous (PRG2: n=27) and control (CTR; n=36) women. Shown are the results of the group*session interaction effect of the general linear model with repeated measures. If significant, the change in between-network connectivity is significantly different across a second pregnancy compared to control women. Tables are color-coded according to F-values, with higher F-values in darker red colours. * $p < 0.05$ after FDR correction for multiple testing. DMN: Default mode network; Cogn-Lang: Cognition/Language network; Perc-Pain: Perception-pain network; Executive: Executive control network.

Supplementary Table 14: Differences in between network connectivity changes across a first and second pregnancy.

	Visual 2	Visual 3	Sensori motor	Visual 1	DMN	Cogn Lang	Audi-tory	Perc Pain	Cere-bellum	Exe-cutive
Visual 2		F=0.22 p=0.89	F=5.25 P=0.32	F=0.13 p=0.92	F=1.04 p = 0.74	F=1.97 P=0.74	F=1.04 p=0.74	F=0.38 p=0.87	F=0.14 p=0.91	F=0.31 p=0.87
Visual 3	F=0.22 p=0.89		F=1.66 p=0.74	F=0.67 p=0.80	F=0.009 p=1.00	F=1.28 p=0.74	F=3.55 p=0.41	F=0.007 p=1.00	F=0.84 p=0.75	F=0.32 p=0.87
Sensori motor	F=5.25 P=0.32	F=1.66 p=0.74		F=2.03 p=0.74	F<0.01 p=1.00	F=1.19 p=0.74	F=13.5 p=0.02 *	F=1.28 p=0.74	F=3.41 p=0.42	F=0.40 p=0.86
Visual 1	F=0.13 p=0.92	F=0.67 p=0.80	F=2.03 p=0.74		F<0.01 p=1.00	F=0.32 p=0.87	F=0.63 p=0.80	F<0.01 p=1.00	F=0.09 p=0.94	F=1.16 p=0.74
DMN	F=1.04 p = 0.74	F=0.009 p=1.00	F<0.01 p=1.00	F<0.01 p=1.00		F=4.13 p=0.40	F<0.01 p=1.00	F=1.04 p=0.74	F=4.59 p=0.36	F=1.80 p=0.74
Cogn-Lang	F=1.97 P=0.74	F=1.28 p=0.74	F=1.19 p=0.74	F=0.32 p=0.87	F=4.13 p =0.40		F=1.41 p=0.74	F=0.26 p=0.89	F=3.86 p=0.40	F=0.22 p=0.89
Audi-tory	F=1.04 p=0.74	F=3.55 p=0.41	F=13.5 p=0.02 *	F=0.63 p=0.80	F<0.01 p=1.00	F=1.41 p=0.74		F=0.29 p=0.88	F=1.08 p=0.74	F=0.19 p=0.90
Perc-Pain	F=0.38 p=0.87	F=0.007 p=1.00	F=1.28 p=0.74	F<0.01 p=1.00	F=1.04 p=0.74	F=0.26 p=0.89	F=0.29 p=0.88		F=1.21 p=0.74	F=0.06 p=0.98
Cere-bellum	F=0.14 p=0.91	F=0.84 p=0.75	F=3.41 p=0.42	F=0.09 p=0.94	F=4.59 p=0.36	F=3.86 p=0.40	F=1.08 p=0.74	F=1.21 p=0.74		F=0.62 p=0.80
Exe-cutive	F=0.31 p=0.87	F=0.32 p=0.87	F=0.40 p=0.86	F=1.16 p=0.74	F=1.80 p=0.74	F=0.22 p=0.89	F=0.19 p=0.90	F=0.06 p=0.98	F=0.62 p=0.80	

Note. We compared between-network connectivity changes (from PRE to POST) between the 10 identified functional networks in multiparous (PRG2: n=27) and primiparous (PRG1: n=40) women. Shown are the results of the group*session interaction effect of the general linear model with repeated measures. If significant, the change in between-network connectivity is significantly different across a second pregnancy compared to a first pregnancy. Tables are color-coded according to F-values, with higher F-

values in darker red colours. * $p < 0.05$ after FDR correction for multiple testing. DMN: Default mode network; Cogn-Lang: Cognition/Language network; Perc-Pain: Perception-pain network; Executive: Executive control network. We found a significant interaction between a first and second pregnancy in between network coherence between the sensorimotor and auditory network, driven by an increase in coherence across a first pregnancy, although this increase was not significantly different from control women.

Supplementary Table 15: Connectivity changes between sensorimotor and auditory network across a first and second pregnancy and in control women.

	PRE	POST	Wilcoxon signed rank test
PRG2	R=0.37 ± 0.22	R=0.26 ± 0.26	V = 250; p=0.80
PRG1	R=0.13 ± 0.30	R=0.32 ± 0.24	V = 165; p=0.03 *
CTR	R=0.28 ± 0.24	R=0.29 ± 0.22	V = 317; p = 1.0

Note. Mean and standard deviation of between network coherence of the sensorimotor and auditory network in multiparous (PRG2; n=27), primiparous (PRG1; n=40) and control (CTR; n=36) women at the pre-pregnancy baseline (PRE) and the postpartum session (POST). Between network coherence is measured as the Pearson correlation between time courses. Results of the two-sided paired t-test within groups are given. * $p < 0.05$ after FDR correction for multiple testing.

Supplementary Table 16: White matter fractional anisotropy changes across a second and first pregnancy and control women.

White matter tract		FA (mean ± SD)				F-test		
			PRG2	PRG1	CTR	PRG2 - CTR	PRG1 - CTR	PRG2 – PRG1
Thal	L	PRE	0.38 ± 0.02	0.38 ± 0.01	0.38 ± 0.01	F(68) = 0.14, p = 0.82	F(78)=0.63, p = 0.53	F(68)=0.08, p = 0.86
		POST	0.38 ± 0.02	0.38 ± 0.01	0.38 ± 0.02			
	R	PRE	0.36 ± 0.02	0.36 ± 0.02	0.36 ± 0.01	F(68) = 0.35, p = 0.69	F(78)=0.12, p = 0.81	F(68)=0.68, p = 0.56
		POST	0.36 ± 0.02	0.35 ± 0.02	0.35 ± 0.01			
CST	L	PRE	0.55 ± 0.02	0.55 ± 0.03	0.55 ± 0.03	F(68) = 0.42, p = 0.68	F(78)=3.00, p = 0.15	F(68)=1.32, p = 0.40
		POST	0.55 ± 0.03	0.55 ± 0.03	0.55 ± 0.03			
	R	PRE	0.57 ± 0.03	0.57 ± 0.03	0.57 ± 0.03	F(68) = 0.75, p = 0.58	F(78)=0.01, p = 0.93	F(68)=0.65, p = 0.56
		POST	0.57 ± 0.03	0.57 ± 0.04	0.57 ± 0.03			
Cing	L	PRE	0.50 ± 0.04	0.50 ± 0.04	0.49 ± 0.04	F(68) = 1.59, p = 0.37	F(78)=1.23, p = 0.42	F(68)=0.02, p = 0.93
		POST	0.49 ± 0.04	0.49 ± 0.05	0.49 ± 0.04			
	R	PRE	0.46 ± 0.05	0.46 ± 0.05	0.45 ± 0.05	F(68) = 0.08, p = 0.86	F(78)=0.02, p = 0.93	F(68)=0.20, p = 0.75
		POST	0.46 ± 0.05	0.45 ± 0.05	0.45 ± 0.05			
Cing Hipp	L	PRE	0.39 ± 0.04	0.39 ± 0.03	0.38 ± 0.03	F(68) = 1.41, p = 0.40	F(78)=1.18, p = 0.41	F(68)=5.17, p = 0.05
		POST	0.41 ± 0.04	0.39 ± 0.03	0.39 ± 0.04			
	R	PRE	0.41 ± 0.04	0.41 ± 0.03	0.40 ± 0.04	F(68) = 0.66, p = 0.59	F(78)=3.58, p = 0.11	F(68)=1.18, p = 0.42
		POST	0.42 ± 0.04	0.41 ± 0.03	0.40 ± 0.03			
Forceps Major		PRE	0.62 ± 0.04	0.62 ± 0.04	0.61 ± 0.03	F(68) = 1.33, p = 0.41	F(78)=0.26, p = 0.70	F(68)=0.42 p = 0.64
		POST	0.63 ± 0.04	0.63 ± 0.04	0.61 ± 0.03			
Forceps Minor		PRE	0.46 ± 0.02	0.47 ± 0.02	0.46 ± 0.02	F(68) = 0.69, p = 0.59	F(78)=1.16, p = 0.41	F(68)=0.003, p = 0.97
		POST	0.46 ± 0.02	0.46 ± 0.02	0.46 ± 0.02			
IFOF	L	PRE	0.48 ± 0.02	0.49 ± 0.02	0.48 ± 0.02	F(68) = 0.02, p = 0.90	F(78)=0.89, p = 0.46	F(68)=1.15, p = 0.42
		POST	0.48 ± 0.02	0.49 ± 0.02	0.48 ± 0.02			
	R	PRE	0.49 ± 0.02	0.49 ± 0.02	0.48 ± 0.02	F(68) = 0.13, p = 0.82	-F(78)=0.99, p = 0.45	F(68)=0.35, p = 0.67
		POST	0.49 ± 0.02	0.49 ± 0.02	0.48 ± 0.02			
ILF	L	PRE	0.47 ± 0.03	0.48 ± 0.02	0.47 ± 0.03	F(68) = 0.18, p = 0.80	F(78)=0.01, p = 0.93	F(68)=0.26, p = 0.72
		POST	0.47 ± 0.02	0.48 ± 0.02	0.47 ± 0.03			
	R	PRE	0.49 ± 0.02	0.49 ± 0.02	0.49 ± 0.03	F(68) = 0.90, p = 0.54	F(78)=0.29, p = 0.69	F(68)=2.90, p = 0.15
		POST	0.49 ± 0.02	0.49 ± 0.02	0.49 ± 0.03			
SLF	L	PRE	0.46 ± 0.03	0.46 ± 0.02	0.46 ± 0.02	F(68) = 3.19, p = 0.15	F(78)=0.93, p = 0.46	F(68)=1.00, p = 0.45
		POST	0.46 ± 0.02	0.46 ± 0.02	0.46 ± 0.02			
	R	PRE	0.46 ± 0.03	0.46 ± 0.02	0.45 ± 0.02	F(68) = 0.04, p = 0.88	F(78)=3.40, p = 0.12	F(68)=3.13, p = 0.14
		POST	0.46 ± 0.03	0.45 ± 0.02	0.45 ± 0.02			
SLFT	L	PRE	0.52 ± 0.06	0.53 ± 0.07	0.50 ± 0.07	F(68) = 0.005, p = 0.98	F(78)=7.86, p = 0.01 *	F(68)=4.09, p = 0.09 #
		POST	0.52 ± 0.07	0.50 ± 0.06	0.50 ± 0.07			
	R	PRE	0.56 ± 0.05	0.55 ± 0.04	0.55 ± 0.05	F(68) = 1.59, p = 0.69	F(78)=0.47, p = 0.60	F(68)=0.50, p = 0.61
		POST	0.55 ± 0.04	0.56 ± 0.05	0.56 ± 0.04			
Unc	L	PRE	0.43 ± 0.03	0.44 ± 0.02	0.43 ± 0.03	F(68) = 0.05, p = 0.88	F(78)=0.04, p = 0.91	F(68)=0.002, p = 0.97
		POST	0.43 ± 0.02	0.44 ± 0.03	0.43 ± 0.03			
	R	PRE	0.46 ± 0.03	0.46 ± 0.04	0.45 ± 0.03	F(68) = 0.52, p = 0.64	F(78)=2.31, p = 0.21	F(68)=03.66, p = 0.10
		POST	0.46 ± 0.03	0.45 ± 0.04	0.45 ± 0.03			

Note. Changes in fractional anisotropy (FA) between the pre-pregnancy (PRE) and early-postpartum (POST) sessions in multiparous (PRG2; n=30), primiparous (PRG1; n=40) and control (CTR; n=40) women. Age-corrected mixed linear models were used to analyse the group * session interaction effects, results are given as F-value and p-value. L = left, R = right. Thal = thalamic radiation, CST = Corticospinal Tract, Cing = Cingulum bundle, Cing Hipp = Cingulum bundle (Hippocampal part), IFOF

= Inferior Fronto-Occipital Fasciculus, ILF = Inferior longitudinal fasciculus, SLF = Superior Longitudinal Fasciculus, SLFT = Superior Longitudinal Fasciculus (Temporal part), Unc = Uncinate Fasciculus. * $p < 0.05$ after FDR-correction for multiple testing, # $p < 0.1$ after FDR-correction for multiple testing.

Supplementary Table 17: White matter mean diffusivity changes across a second and first pregnancy and control women.

White matter tract			MD (mean ± SD)			F-test		
			PRG2	PRG1	CTR	PRG2 - CTR	PRG1 - CTR	PRG2 – PRG1
Thal	L	PRE	77.1 ± 2.4	76.7 ± 2.2	76.6 ± 1.9	F(68) = 0.49, p = 0.60	F(78)=0.001, p = 0.99	F(68)=0.53, p = 0.62
		POST	76.5 ± 2.4	76.4 ± 2.5	76.4 ± 2.5			
	R	PRE	78.5 ± 2.6	77.5± 2.3	77.6± 1.9	F(68) = 0.41, p = 0.61	F(78)=0.03, p = 0.92	F(68)=0.16, p = 0.81
		POST	78.5 ± 2.3	77.7± 2.2	77.9 ± 2.2			
CST	L	PRE	74.2 ± 1.8	73.3 ± 2.0	73.6 ± 2.5	F(68) = 0.65, p = 0.59	F(78)=0.99, p = 0.53	F(68)=3.33, p = 0.13
		POST	73.1 ± 1.9	72.9 ± 1.9	72.8 ± 2.2			
	R	PRE	73.1 ± 2.2	72.2 ± 2.0	72.1± 2.1	F(68) = 6.28, p = 0.03 *	F(78)=0.01, p = 0.96	F(68)=7.44, p = 0.01 *
		POST	71.1 ± 1.7	72.0 ± 2.3	72.0 ± 1.9			
Cing	L	PRE	73.7 ± 2.2	74.2 ± 1.8	74.6± 2.6	F(68) = 0.01, p = 0.93	F(78)=0.04, p = 0.92	F(68)=0.003, p = 0.98
		POST	72.9 ± 2.6	73.3± 2.0	73.8 ± 2.6			
	R	PRE	74.5 ± 2.1	75.5 ± 1.8	75.5± 2.8	F(68) = 0.54, p = 0.60	F(78)=0.10 p = 0.87	F(68)=0.16, p = 0.81
		POST	73.7 ± 2.5	74.9 ± 2.3	75.1 ± 2.8			
Cing Hipp	L	PRE	81.9 ± 3.3	82.9 ± 2.8	84.0 ± 5.3	F(68) = 1.43, p = 0.40	F(78)=0.53, p = 0.70	F(68)=0.34, p = 0.71
		POST	81.4 ± 5.2	81.7± 3.9	82.3 ± 4.9			
	R	PRE	82.3 ± 2.5	81.9 ± 2.6	82.9 ± 3.2	F(68) = 1.17, p = 0.42	F(78)=0.35, p = 0.79	F(68)=2.61, p = 0.19
		POST	80.6 ± 2.6	81.4 ± 3.0	82.0 ± 2.6			
Forceps Major		PRE	81.0 ± 2.4	81.9 ± 2.6	81.9 ± 2.7	F(68) = 0.18, p = 0.77	F(78)=0.53, p = 0.70	F(68)=0.07, p = 0.90
		POST	80.4 ± 2.9	80.3± 2.2	81.4 ± 3.0			
Forceps Minor		PRE	76.6 ± 2.2	81.1 ± 2.3	78.3 ± 2.2	F(68) = 1.90, p = 0.32	F(78)=0.11, p = 0.87	F(68)=1.09, p = 0.45
		POST	76.2 ± 2.4	76.6 ± 2.4	77.4 ± 2.7			
IFOF	L	PRE	76.7± 2.2	77.0 ± 1.5	77.7 ± 2.0	F(68) = 0.12, p = 0.78	F(78)=0.14, p = 0.87	F(68)=0.57, p = 0.62
		POST	76.4 ± 2.2	76.4 ± 1.5	77.3 ± 2.1			
	R	PRE	77.1 ± 1.8	77.1 ± 1.8	78.1 ± 1.7	F(68) = 0.14, p = 0.78	F(78)=0.97, p = 0.53	F(68)=0.27, p = 0.74
		POST	76.9 ± 2.0	77.0 ± 1.8	77.7 ± 1.9			
ILF	L	PRE	77.8 ± 2.2	77.8 ± 1.8	79.2 ± 2.3	F(68) = 0.46, p = 0.60	F(78)=0.11, p = 0.87	F(68)=1.15, p = 0.45
		POST	77.4 ± 2.5	77.1 ± 1.7	78.6 ± 2.5			
	R	PRE	76.3 ± 2.4	76.2 ± 2.0	77.3 ± 1.9	F(68) = 0.008, p = 0.93	F(78)=1.05, p = 0.53	F(68)=0.84, p = 0.52
		POST	75.7 ± 2.2	75.9 ± 1.9	76.7 ± 2.3			
SLF	L	PRE	70.9± 2.2	70.8 ± 1.5	71.4 ± 2.2	F(68) = 0.59, p = 0.60	F(78)=1.07, p = 0.53	F(68)=0.03, p = 0.93
		POST	70.4 ± 2.0	70.3 ± 1.5	71.2 ± 2.2			
	R	PRE	70.1 ± 2.1	70.3 ± 1.8	71.0± 2.2	F(68) = 1.35, p = 0.40	F(78)=0.006, p = 0.96	F(68)=1.88, p = 0.29
		POST	69.4 ± 2.2	70.0 ± 1.7	70.7 ± 2.4			
SLFT	L	PRE	76.2 ± 3.3	75.9± 5.3	77.1± 4.3	F(68) = 0.47, p = 0.60	F(78)=0.34, p = 0.79	F(68)=0.01, p = 0.97
		POST	75.6 ± 4.2	75.3 ± 4.6	77.0 ± 4.0			
	R	PRE	74.1± 3.0	73.5 ± 2.6	74.8± 3.0	F(68) = 0.02, p = 0.93	F(78)=0.14, p = 0.87	F(68)=0.04, p = 0.92
		POST	73.6 ± 3.2	73.1 ± 2.8	74.2 ± 3.0			
Unc	L	PRE	79.9 ± 2.5	79.6 ± 2.5	80.9± 2.3	F(68) = 1.60, p = 0.37	F(78)=0.19, p = 0.87	F(68)=0.73, p = 0.56
		POST	80.2 ± 2.9	79.4 ± 2.4	80.5 ± 2.3			
	R	PRE	78.4 ± 2.3	78.7 ± 2.2	80.1 ± 2.0	F(68) = 0.90, p = 0.50	F(78)=0.04, p = 0.92	F(68)=1.23, p = 0.44
		POST	78.9 ± 2.6	78.7 ± 2.6	80.1 ± 2.4			

Note. Changes in mean diffusivity (MD) between the pre-pregnancy (PRE) and early-postpartum (POST) sessions in multiparous (PRG2; n=30), primiparous (PRG1; n=40) and control (CTR; n=40) women, given as 10^{-5} mm^2/s. Age-corrected mixed linear models were used to analyse the group * session interaction effects, results are given as F-value and p-value. L = left, R = right. Thal = thalamic radiation, CST = Corticospinal Tract, Cing = Cingulum bundle, Cing Hipp = Cingulum bundle (Hippocampal part), IFOF = Inferior Fronto-Occipital Fasciculus, ILF = Inferior longitudinal fasciculus, SLF = Superior Longitudinal Fasciculus, SLFT = Superior

Longitudinal Fasciculus (Temporal part), Unc = Uncinate Fasciculus. * $p < 0.05$ after FDR-correction for multiple testing, # $p < 0.1$ after FDR-correction for multiple testing.

Supplementary Table 18: Metabolite concentration differences across a second pregnancy and control women.

Metabolite	Metabolite concentrations (mean ± SD)			F	p
		PRG2	CTR		
tNAA	PRE	11.322 ± 0.476	11.232 ± 0.435	0.030	0.86
	POST	11.461 ± 0.387	11.394 ± 0.438		
tCho	PRE	1.210 ± 0.138	1.199 ± 0.147	1.86	0.18
	POST	1.364 ± 0.146	1.288 ± 0.166		
tCr	PRE	7.525 ± 0.493	7.506 ± 0.456	4.24	0.04
	POST	8.129 ± 0.496	7.781 ± 0.461		
Glu	PRE	9.461 ± 0.485	9.905 ± 0.708	0.105	0.75
	POST	9.816 ± 0.651	10.188 ± 0.784		
Ins	PRE	4.375 ± 0.412	4.330 ± 0.522	1.73	0.19
	POST	4.734 ± 0.541	4.523 ± 0.489		

Note. Changes in metabolite concentrations (mM) between the pre-pregnancy (PRE) and early-postpartum (POST) sessions in multiparous (PRG2; n=27) and control (CTR; n=37) women. Age-corrected mixed linear models were used to analyse the group * session interaction effects, results are given as F-value and p-value. These effects do not survive the FDR-correction for multiple testing. tNAA = Nacetylaspartate (including contributions from N-acetylaspartylglutamate), Cho = Choline (phosphorylcholine and glycerophosphorylcholine), tCr = Creatine (creatine and phosphocreatine), Glu = Glutamate, Ins = myo-Inositol.

Supplementary Table 19: Metabolite concentration differences across a first and second pregnancy.

Metabolite	Metabolite concentrations (mean ± SD)			F	p
		PRG2	PRG1		
tNAA	PRE	11.322 ± 0.476	11.178 ± 0.41	0.177	0.66
	POST	11.461 ± 0.387	11.376 ± 0.564		
tCho	PRE	1.210 ± 0.138	1.206 ± 0.124	0.258	0.61
	POST	1.364 ± 0.146	1.380 ± 0.127		
tCr	PRE	7.525 ± 0.493	7.497 ± 0.511	0.187	0.67
	POST	8.129 ± 0.496	8.040 ± 0.406		
Glu	PRE	9.461 ± 0.485	9.674 ± 0.682	0.042	0.84
	POST	9.816 ± 0.651	9.989 ± 0.641		
Ins	PRE	4.375 ± 0.412	4.377 ± 0.479	0.005	0.94
	POST	4.734 ± 0.541	4.746 ± 0.539		

Note. Changes in metabolite concentrations (mM) between the pre-pregnancy (PRE) and early-postpartum (POST) sessions in multiparous (PRG2; n=27) and primiparous (PRG1; n=39) women. Age-corrected mixed linear models were used to analyse the group * session interaction effects, results are given as F-value and p-value. These effects do not survive the FDR-correction for multiple testing. tNAA = Nacetylaspartate (including contributions from N-acetylaspartylglutamate), Cho = Choline (phosphorylcholine and glycerophosphorylcholine), tCr = Creatine (creatine and phosphocreatine), Glu = Glutamate , Ins = myo-Inositol.

Supplementary Table 20: Demographic information of the study groups.

Characteristic	PRG2 (n=30)	PRG1 (n=40)	CTR (n=40)	Group differences
Age at PRE session (M ± SD years)	32.03 ± 2.33	29.35 ± 3.51	29.33 ± 3.57	F(2,107) = 7.476, p = 0.001<br><br><u>PRG2 vs PRG1:</u><br>Diff = 2.68, CI (0.77 – 4.59), p = 0.0027*<br><br><u>CTRL vs PRG2:</u><br>Diff = -2.71, CI (-4.62 – -0.80), p = 0.0025*<br><br><u>CTR vs PRG1</u><br>Diff = -0.03, CI (-1.80 – 1.75), p = 1.00
Education (M ± SD Verhage score)	6.33 ± 0.76	6.43 ± 0.78	6.65 ± 0.53	F(2,107) = 2.00, p = 0.14
Time between PRE and POST (M ± SD days)	502.70 ± 125.02	509.78 ± 158.81	457.95 ± 81.95	F(2,107) = 1.93, p = 0.15
Time between birth and POST (M ± SD days)	80.32 ± 27.72	100.63 ± 70.81	-	F(1,66) = 2.07, p = 0.16
Time between birth and POST1 (M ± SD days)	408.67 ± 38.75	402.58 ± 64.76	-	F(1,30) = 0.05, p = 0.83
Age of first child at PRE (days)	695.04 ± 343.54	-	-	
Natural conception	29	37	-	
Assisted conception	1	3	-	
Vaginal birth	20	31	-	
Caesarean section	7	9	-	
Breastfeeding	21	30	-	
Formula feeding	7	10	-	
Twins	0	1	-	

Note. Demographic information of the studied groups and between-group differences. PRG2 = multiparous women (n = 30); PRG1 = primiparous women (n = 40), CTR = nulliparous control women (n = 40). M = mean, SD = standard deviation. Diff = difference between the two groups, CI = confidence interval. Test results of ANOVAs and two-sided t-tests are shown. * p < 0.05 after Bonferroni correction in post-hoc tests.

Supplementary Table 21: Freesurfer's Euler number as a measure of T1w image quality per group and per timepoint.

Group	PRE	POST	POST1
PRG2	-27.03 ± 11.42	-26.33 ± 9.49	-26.43 ± 8.49
PRG1	-30.30 ± 13.91	-30.13 ± 11.44	-27.86 ± 11.70
CTR	-26.90 ± 11.84	-29.78 ± 11.38	-30.30 ± 11.44

Note. Mean ± standard deviation of Freesurfer's Euler number per group before pregnancy (PRE), in the early postpartum period (POST) and in the late postpartum period (POST1). PRG2 = multiparous women (n = 30), PRG1 = primiparous women (n = 40), CTR = nulliparous women (n = 40).

Supplementary Table 22: Overview of head motion during the resting-state fMRI per group and per timepoint.

Motion	PRG2		PRG1		CTRL		Group * Time
	PRE	*POST*	*PRE*	*POST*	*PRE*	*POST*	interaction
Mean FD	0.09 ± 0.03	0.10 ± 0.04	0.09 ± 0.03	0.09 ± 0.03	0.10 ± 0.03	0.08 ± 0.03	F = 1.19, p = 0.31
Max Tx	0.10 ± 0.06	0.09 ± 0.07	0.10 ± 0.07	0.10 ± 0.06	0.10 ± 0.04	0.10 ± 0.07	F=0.21, p = 0.81
Max Ty	0.30 ± 0.19	0.29 ± 0.14	0.29 ± 0.28	0.30 ± 0.16	0.29 ± 0.16	0.24 ± 0.09	F=0.66, p = 0.52
Max Tz	0.35 ± 0.15	0.29 ± 0.17	0.36 ± 0.29	0.30 ± 0.20	0.40 ± 0.26	0.31 ± 0.19	F=0.22, p = 0.80
Max Rx	0.41 ± 0.33	0.33 ± 0.19	0.38 ± 0.25	0.42 ± 0.36	0.47 ± 0.38	0.35 ± 0.20	F=1.63, p = 0.20
Max Ry	0.19 ± 0.09	0.22 ± 0.15	0.18 ± 0.12	0.16 ± 0.10	0.22 ± 0.13	0.18 ± 0.10	F=1.33, p = 0.27
Max Rz	0.14 ± 0.09	0.14 ± 0.09	0.11 ± 0.06	0.11 ± 0.08	0.14 ± 0.08	0.17 ± 0.15	F=0.44, p = 0.64

Note. Different measures of head motion during resting-state fMRI measured per group and time point, without the excluded participants with too much head motion. Mixed linear models were used to analyse the group * time interaction effects, results are given as F-value and p-value. PRG2 = multiparous women (n = 30), PRG1 = primiparous women (n = 40), CTR = nulliparous control women (n = 40). PRE = before pregnancy, POST = in the early postpartum period. Mean FD = mean frame-wise displacement, Max Tx = maximum translation in the x-direction, Max Ty = maximum translation in the y-direction, Max Tz = maximum translation in the z-direction, Max Rx = maximum rotation along the x-direction, Max Ry = maximum rotation along the y-direction and Max Rz = maximum rotation along the z-direction.

References

1. Yeo, B. T. T. *et al*. The organization of the human cerebral cortex estimated by intrinsic functional connectivity. *J. Neurophysiol*. **106**, 1125–1165 (2011).
