# Supplementary material for: The effects of a second pregnancy on women’s brain structure and function
Source: Nat Commun. 2026 Feb 19;17:1495. doi: 10.1038/s41467-026-69370-8 (PMC12920768; doi:10.1038/s41467-026-69370-8)
Supplement: Supplementary file 2 — Reporting Summary [file 41467_2026_69370_MOESM2_ESM.pdf]

nature portfolio

Corresponding author(s): Milou Straathof
Elseline Hoekzema

Last updated by author(s): Jan 22, 2026

Reporting Summary

Nature Portfolio wishes to improve the reproducibility of the work that we publish. This form provides structure for consistency and transparency in reporting. For further information on Nature Portfolio policies, see our Editorial Policies and the Editorial Policy Checklist.

Please do not complete any field with "not applicable" or n/a. Refer to the help text for what text to use if an item is not relevant to your study.
For final submission: please carefully check your responses for accuracy; you will not be able to make changes later.

Statistics

For all statistical analyses, confirm that the following items are present in the figure legend, table legend, main text, or Methods section.

n/a	Confirmed	
☐	☒	The exact sample size (*n*) for each experimental group/condition, given as a discrete number and unit of measurement
☐	☒	A statement on whether measurements were taken from distinct samples or whether the same sample was measured repeatedly
☐	☒	The statistical test(s) used AND whether they are one- or two-sided<br>*Only common tests should be described solely by name; describe more complex techniques in the Methods section.*
☐	☒	A description of all covariates tested
☐	☒	A description of any assumptions or corrections, such as tests of normality and adjustment for multiple comparisons
☐	☒	A full description of the statistical parameters including central tendency (e.g. means) or other basic estimates (e.g. regression coefficient) AND variation (e.g. standard deviation) or associated estimates of uncertainty (e.g. confidence intervals)
☐	☒	For null hypothesis testing, the test statistic (e.g. *F*, *t*, *r*) with confidence intervals, effect sizes, degrees of freedom and *P* value noted<br>*Give P values as exact values whenever suitable.*
☒	☐	For Bayesian analysis, information on the choice of priors and Markov chain Monte Carlo settings
☒	☐	For hierarchical and complex designs, identification of the appropriate level for tests and full reporting of outcomes
☐	☒	Estimates of effect sizes (e.g. Cohen's *d*, Pearson's *r*), indicating how they were calculated

Our web collection on statistics for biologists contains articles on many of the points above.

Software and code

Policy information about availability of computer code

Data collection: No specific software was used during data

Data analysis: Anatomical MRI:
Processing T1-weighted MRI images: SPM (version SPM12), implemented in Matlab 7.8, I
7.2.0).
Multivariate classification analyses: PRonTo (v3.0).
Statistical analyses & plots: Rstudio 2023.06.1.
Diffusion-weighted MRI: processing: MRTrix3, FSL (version 6.0.3), statistical analys
Proton Magnetic Resonance Spectroscopy: processing: LCModel version 6.3-1M; FSL 5.0.

For manuscripts utilizing custom algorithms or software that are central to the research but not yet described in published literature, software must be made available to editors and reviewers. We strongly encourage code deposition in a community repository (e.g. GitHub). See the Nature Portfolio guidelines for submitting code & software for further information.

Data

Policy information about availability of data

All manuscripts must include a data availability statement. This statement should provide the following information, where applicable:
- Accession codes, unique identifiers, or web links for publicly available datasets
- A description of any restrictions on data availability
- For clinical datasets or third party data, please ensure that the statement adheres to our policy

Source data for each figure are provided with this paper and in FigShare (DOI: https://doi.org/10.6084/m9.figshare.31144273). The raw MRI data and group/demographic information generated in this study for the participants who have provided permission to share their data have been deposited in the Open

Source data for each figure are provided with this paper and in FigShare (DOI: https://doi.org/10.6084/m9.figshare.31144273). The raw MRI data and

Research involving human participants, their data, or biological material

Policy information about studies with human participants or human data. See also policy information about sex, gender (identity/presentation), and sexual orientation and race, ethnicity and racism.

Reporting on sex and gender: We only included self-reported women in this study. The biological cor

Reporting on race, ethnicity, or other socially relevant groupings: We used the self-reported level

Population characteristics: The relevant variables for our study included age, medical history, concepti 0.53).

Recruitment: Participants were recruited using advertisements, flyers (at local GPs and pharmacies)

Ethics oversight: Ethics Review Board of the Leiden University Medical

Note that full information on the approval of the study protocol must also be provided in the manuscript.

Field-specific reporting

Please select the one below that is the best fit for your research. If you are not sure, read the appropriate sections before making your selection.

☐ Life sciences ☒ Behavioural & social sciences ☐ Ecological, evolutionary & environmental sciences

For a reference copy of the document with all sections, see nature.com/documents/nr-reporting-summary-flat.pdf

Behavioural & social sciences study design

All studies must disclose on these points even when the disclosure is negative.

Study description: Quantitative longitudinal prospective cohort

Research sample: This concerns a prospective cohort study in which women participated who wanted to

Sampling strategy: The sampling procedure involved convenience sampling with observational (non-ran

Data collection: MRI data: Philips 3T MRI scanner
Questionnaires: Qualtrics
The researchers were not blinded to the experimental condition given that it was c

Timing: The project started in January 2015 and ended in March 2020.Data collection then ended prem

Data exclusions: All collected anatomical and DTI scans were visually checked and could be used.
Seven subject had to be excluded from the MRS analyses due to a displacement of th
For the resting state analyses, 4 participants from the CTR group and 3 participan

Non-participation: Four women that did not experience a second pregnancy during the course of the s

Randomization: Randomization is not applicable to this study, since group allocation depended on th

Reporting for specific materials, systems and methods

We require information from authors about some types of materials, experimental systems and methods used in many studies. Here, indicate whether each material, system or method listed is relevant to your study. If you are not sure if a list item applies to your research, read the appropriate section before selecting a response.

Materials & experimental systems

n/a	Involved in the study	
☒	☐	Antibodies
☒	☐	Eukaryotic cell lines
☒	☐	Palaeontology and archaeology
☒	☐	Animals and other organisms
☒	☐	Clinical data
☒	☐	Dual use research of concern
☒	☐	Plants

Methods

n/a	Involved in the study	
☒	☐	ChIP-seq
☒	☐	Flow cytometry
☐	☒	MRI-based neuroimaging

Antibodies

Antibodies used: Describe all antibodies used in the study; as applicable, provide supplier name, c

Validation: Describe the validation of each primary antibody for the species and application, notin

Eukaryotic cell lines

Policy information about cell lines and Sex and Gender in Research

Cell line source(s): State the source of each cell line used and the sex of all primary cell lines

Authentication: Describe the authentication procedures for each cell line used OR declare that none

Mycoplasma contamination: Confirm that all cell lines tested negative for mycoplasma contamination

nature portfolio | reporting summary
April 2023

Commonly misidentified lines
(See ICLAC register) | Name any commonly misidentified cell lines used in the study and provide a rationale for their use.

Palaeontology and Archaeology

Specimen provenance | Provide provenance information for specimens and describe permits that were ob

Specimen deposition | Indicate where the specimens have been deposited to permit free access by othe

Dating methods | If new dates are provided, describe how they were obtained (e.g. collection, storag

☐ Tick this box to confirm that the raw and calibrated dates are available in the paper or in Supplementary Information.

Ethics oversight | Identify the organization(s) that approved or provided guidance on the study prot

Note that full information on the approval of the study protocol must also be provided in the manuscript.

Animals and other research organisms

Policy information about studies involving animals; ARRIVE guidelines recommended for reporting a

Laboratory animals | For laboratory animals, report species, strain and age OR state that the study

Wild animals | Provide details on animals observed in or captured in the field; report species and a

Reporting on sex | Indicate if findings apply to only one sex; describe whether sex was considered i

Field-collected samples | For laboratory work with field-collected samples, describe all relevant pa

Ethics oversight | Identify the organization(s) that approved or provided guidance on the study prot

Note that full information on the approval of the study protocol must also be provided in the manuscript.

Clinical data

Policy information about clinical studies

All manuscripts should comply with the ICMJE guidelines for publication of clinical research and a completed CONSORT checklist must be included with all submissions.

Clinical trial registration | Provide the trial registration number from ClinicalTrials.gov or an eq

Study protocol | Note where the full trial protocol can be accessed OR if not available, explain

Data collection | Describe the settings and locales of data collection, noting the time periods of r

Outcomes | Describe how you pre-defined primary and secondary outcome measures and how you assessed

Dual use research of concern

Policy information about dual use research of concern

Hazards

Could the accidental, deliberate or reckless misuse of agents or technologies generated in the work, or the application of information presented in the manuscript, pose a threat to:

No	Yes	
☐	☐	Public health
☐	☐	National security
☐	☐	Crops and/or livestock
☐	☐	Ecosystems
☐	☐	Any other significant area

Experiments of concern

Does the work involve any of these experiments of concern:

No	Yes	
☐	☐	Demonstrate how to render a vaccine ineffective
☐	☐	Confer resistance to therapeutically useful antibiotics or antiviral agents
☐	☐	Enhance the virulence of a pathogen or render a nonpathogen virulent
☐	☐	Increase transmissibility of a pathogen
☐	☐	Alter the host range of a pathogen
☐	☐	Enable evasion of diagnostic/detection modalities
☐	☐	Enable the weaponization of a biological agent or toxin
☐	☐	Any other potentially harmful combination of experiments and agents

Plants

ChIP-seq

Data deposition

Confirm that both raw and final processed data have been deposited in a public database such as GEO.

Confirm that you have deposited or provided access to graph files (e.g. BED files) for the called peaks.

Data access links
May remain private before publication.
For "Initial submission" or "Revised version" documents, provide revie
data

Files in database submission
Provide a list of all files available in the database

Genome browser session (e.g. UCSC)
Provide a link to an anonymized genome browser session for "Initial submission" and "Revised version" documents only, to enable peer review. Write "no longer applicable" for "Final submission" documents.

Methodology

Replicates
Describe the experimental replicates, specifying number, type and replicate

Sequencing depth
Describe the sequencing depth for each experiment, providing the total number of

Antibodies
Describe the antibodies used for the ChIP-seq experiments; as applicable, provide supplier name, catalog number, clone name, and lot number.

Peak calling parameters
Specify the command line program and parameters used for read mapping and

Data quality
Describe the methods used to ensure data quality in full detail, including how many p

Software
Describe the software used to collect and analyze the ChIP-seq data. For custom code that

Flow Cytometry

Plots

Confirm that:

The axis labels state the marker and fluorochrome used (e.g. CD4-FITC).

The axis scales are clearly visible. Include numbers along axes only for bottom left plot of group (a 'group' is an analysis of identical markers).

All plots are contour plots with outliers or pseudocolor plots.

A numerical value for number of cells or percentage (with statistics) is provided.

Methodology

Sample preparation
Describe the sample preparation, detailing the biological source of the cells a

Instrument
Identify the instrument used for data collection, specifying make and model

Software
Describe the software used to collect and analyze the flow cytometry data. For custom coc

Cell population abundance
Describe the abundance of the relevant cell populations within post-sort

Gating strategy
Describe the gating strategy used for all relevant experiments, specifying the pre

Tick this box to confirm that a figure exemplifying the gating strategy is provided in the Supplementary Information.

Magnetic resonance imaging

Experimental design

Design type
resting-

Design specifications
n/a (resting state, fixation

Behavioral performance measures
n/

Acquisition

Imaging type(s)
structural, diffusion, MR spectroscopy, resting-state

Field strength
3

Sequence & imaging parameters
Structural: 3D T1-weighted images were acquired (Repetition Time (TR
Resting-state functional: T2*-weighted whole-brain echo-planar image
slices.
Diffusion: fast MRI (SENSE) factor = 3; Flip Angle 90°; 75 slices of

Area of acquisition
Whole-brain, except for spectroscopy which included 2 volumes of interest (Pre

Diffusion MRI
☒ Used ☐ Not used

Preprocessing

Preprocessing software
Structural: Freesurfer 7.2.0; longitudinal recon-all pipeline, 10 mm FWHM s
SPM12 in Matlab 7.8: longitudinal symmetric diffeomorphic modeling pipeline
kernel.
Diffusion: MRTrix3: FSL 6.0: dwidenoise, dwifslpreproc and dwibiascorrect.
Resting-state functional: DPARSF V4.5: slice time correction, realignment,

Normalization
Structural: Freesurfer: non-linear registration, 10 mm FWHM smoothing kernel.
Structural: SPM: DARTEL (non-linear), smoothed with 10-mm FWHM smoothing kernel for
PRonTo.
Diffusion: FSL v 6.0.3 (non-linear)

Spectroscopy: no normalization, quantification within individual volume of interest.

Normalization template
Structural: Freesurfer: fsaverage

Structural: SPM: MNI space for
PRonTo
Diffusion: JHU-ICBM atlas template
Functional: MNI space

Noise and artifact removal Subjects with frame-wise displacement (FD) exceeding 2mm (for translati

Volume censoring n/

Statistical modeling & inference

Model type and settings Group spatial independent component analysis

Effect(s) tested n/

Specify type of analysis: ☐ Whole brain ☐ ROI-based ☒ Both

Statistic type for inference
(See Eklund et al. 2016)

Specify voxel-wise or cluster-wise and report all relevant parameters for cluster-wise methods.

Correction Structural: FDR-corrected across the brain
Diffusion: FDR-corrected across white matter tracts of interest
Functional: FWE-corrected across the whole brain within independent components; FDR-cor
networks

Models & analysis

n/a	Involved in the study
☐	☒ Functional and/or effective connectivity
☒	☐ Graph analysis
☒	☐ Multivariate modeling or predictive analysis

Functional and/or effective connectivity Between-network connectivity (Pearson's)

Graph analysis Report the dependent variable and connectivity measure, specifying weighted graph c

Multivariate modeling and predictive analysis Specify independent variables, features extraction a

This checklist template is licensed under a Creative Commons Attribution 4.0 International License, which permits use, sharing, adaptation, distribution and reproduction in any medium or format, as long as you give appropriate credit to the original author(s) and the source, provide a link to the Creative Commons license, and indicate if changes were made. The images or other third party material in this article are included in the article's Creative Commons license, unless indicated otherwise in a credit line to the material. If material is not included in the article's Creative Commons license and your intended use is not permitted by statutory regulation or exceeds the permitted use, you will need to obtain permission directly from the copyright holder. To view a copy of this license, visit http://creativecommons.org/licenses/bv/4.0/
